# Editorial: Impacts of 2021 WHO classification on the precise diagnosis and management of gliomas

**DOI:** 10.3389/fnins.2024.1366523

**Published:** 2024-02-21

**Authors:** Xiaopeng Guo, Yu Wang, Wenbin Ma

**Affiliations:** Department of Neurosurgery, Peking Union Medical College Hospital, Chinese Academy of Medical Sciences and Peking Union Medical College, Beijing, China

**Keywords:** WHO classification of central nervous system tumors, glioma, molecular alteration, diagnosis, treatment

Gliomas represent the most prevalent and lethal primary tumors within the central nervous system (CNS), exhibiting considerable histological and molecular diversity. Histologically, gliomas are categorized into four grades, with grade-4 tumors displaying the highest growth rate, aggressive behavior, and contributing to the most unfavorable prognosis. Notably, gliomas of the same grades may exhibit distinct clinical presentations based on differing molecular alterations (Louis et al., 2021). For example, low-grade IDH-wildtype astrocytic gliomas with *pTERT* mutation, *EGFR* amplification, or chromosomal +7/−10 changes demonstrate significantly more aggressive features and a poorer prognosis than their low-grade counterparts lacking such molecular alterations.

Building upon the 4th edition of the WHO classification of CNS tumors (WHO CNS4) and subsequent research by cIMPACT-NOW, the WHO CNS5 classification was unveiled in 2021. This classification integrates both histopathologic and molecular considerations to enhance the diagnostic classification and grading system of CNS tumors, aiding clinicians in determining optimal therapies (Wen and Packer, 2021). The WHO CNS5 classification categorizes gliomas, glioneuronal tumors, and neuronal tumors into six groups: adult-type diffuse gliomas, pediatric-type diffuse low-grade gliomas, pediatric-type diffuse high-grade gliomas, circumscribed astrocytic gliomas, glioneuronal and neuronal tumors, and ependymal tumors (Horbinski et al., 2022). Adult-type diffuse gliomas, including astrocytoma, oligodendroglioma, and glioblastoma, constitute the majority of gliomas and have undergone significant changes from the WHO CNS4 to the WHO CNS5 classification. Under the current classification, low-grade IDH-mutant astrocytoma is upgraded to a grade-4 astrocytoma upon CDKN2A/B homozygous deletion. Similarly, low-grade IDH-wildtype astrocytic gliomas are re-classified as grade-4 glioblastomas if any one of the three genetic parameters is present: *pTERT* mutation, *EGFR* amplification, or chromosomal +7/−10 changes. Pediatric-type diffuse gliomas are newly incorporated entities in the current classification, characterized by distinct molecular alterations involving *MYB, MYB-L1, BRAF, H3K27, H3G34, MAPK* pathway, and *NTRK* family (Cohen, 2022; Horbinski et al., 2022). These molecular markers, although making precise diagnosis challenging and optimal treatments unclear, offer valuable insights for potential targeted therapies.

The significant revisions to the WHO classification of CNS tumors have profound implications for the diagnosis and treatment of gliomas. This Research Topic comprises a collection of articles that delve into the transformations introduced by the WHO CNS5 classification in the clinical diagnosis and treatment of gliomas. A brief overview of the accepted articles in this Topic is presented below. Guo et al. re-examined the genetic alterations of IDH-wildtype gliomas in a single institute, shedding light on clinical, radiological, molecular, and survival characteristics of histological (hist-GBM) and molecular (mol-GBM) subtypes under the new classification. The study addressed the scarcity of knowledge on their features in the real-world settings. Wang et al. analyzed data from 98 elderly GBM patients and identified *KRAS* and *PPM1D* as unique prognostic values. The study explored prognostic markers crucial for this population. Chen et al. investigated the clinical characteristics, radiological features, molecular alterations, and survival profile of 20 adult patients with pediatric-type gliomas, contributing to the understanding of this unique patient group. *KMT5B* and *MET* were identified as determinants for survival and potential therapeutic targets. Ge et al. and Zhang et al. explored the structural and functional changes of the brain in patients with gliomas involving frontal and temporal lobes, providing insights into the protective mechanisms of the human brain in the context of gliomas. Song et al. investigated the role of *ARSD* in gliomas, revealing its promotion of tumor development by inducing an immunosuppressive tumor microenvironment, modulating immune cell infiltration, affecting the cancer-immunity cycle, and regulating the *JAK2*/*STAT3* pathway, providing potential therapeutic targets for glioma treatment.

Although accurate classification of glioma is crucial for optimal treatment and improved patient survival, challenges still persist (van den Bent et al., 2023). Molecular testing is not universally performed in daily clinical practice, leading to only histology-based diagnoses in a significant number of cases. Despite the WHO CNS5 classification, some gliomas are still labeled as “not elsewhere classified” (NEC) even after comprehensive genetic evaluation, prompting the need for further revisions of the current classification. Although targeted therapy has shown promise for certain mutations like *BRAF*, the majority of gliomas are still treated only with surgery, radiotherapy, and temozolomide. Further application of the WHO CNS5 classification are expected to facilitate the enrollment of more homogeneous patients into specific clinical trials, enhancing scientific rigor of the trials and promoting patient survival.

## Author contributions

XG: Conceptualization, Writing—original draft, Writing—review & editing. YW: Conceptualization, Supervision, Writing—review & editing. WM: Conceptualization, Supervision, Writing—review & editing.
